# Efficacy of paracetamol added to WHO step III opioids in chronic pain control: study protocol for a randomised, double-blind, placebo-controlled, non-inferiority, multicentre study in Switzerland

**DOI:** 10.1136/bmjopen-2025-107360

**Published:** 2025-12-31

**Authors:** Christina Kotoula, Maria M Wertli, Konrad Streitberger, Sacha I Rothschild, Andreas Limacher, Felix Hammann, Stephan Krähenbühl, Manuel Haschke, Evangelia Liakoni

**Affiliations:** 1 Clinical Pharmacology and Toxicology, Department of General Internal Medicine, Inselspital, Bern University Hospital, University of Bern, Bern, Switzerland; 2 Graduate School for Health Sciences, University of Bern, Bern, Switzerland; 3 Department of General Internal Medicine, Inselspital, Bern University Hospital, University of Bern, Bern, Switzerland; 4 Department of Internal Medicine, Cantonal Hospital Baden, Baden, Switzerland; 5 Department of Anaesthesiology and Pain Medicine, Inselspital, Bern University Hospital, University of Bern, Bern, Switzerland; 6 Center of Oncology/Hematology, Cantonal Hospital Baden, Baden, Switzerland; 7 Medical Faculty, University of Basel, Basel, Switzerland; 8 Department of Infectious Diseases, Inselspital, Bern University Hospital, University of Bern, Bern, Switzerland; 9 Functioning Information Reference Lab, Swiss Paraplegic Research, Nottwil, Switzerland; 10 Division of Clinical Pharmacology & Toxicology, University Hospital Basel, Basel, Switzerland

**Keywords:** Clinical Trial, Randomized Controlled Trial, Chronic Pain

## Abstract

**Introduction:**

The analgesic and antipyretic paracetamol (acetaminophen) is generally considered safe in therapeutic doses. The most important toxic effect is hepatotoxicity after supratherapeutic doses or in the presence of risk factors (eg, malnutrition, alcoholism). According to the WHO analgesic ladder, a combination of a non-opioid analgesic such as paracetamol with a strong opioid is recommended as step III treatment of patients with chronic pain, despite limited evidence for this approach. The main aim of this study is to test the hypothesis that paracetamol does not provide clinically relevant benefits when added to strong opioids in patients with chronic pain.

**Methods and analysis:**

Investigator-initiated, randomised, double-blind, placebo-controlled, non-inferiority trial at two Swiss hospitals. A total of 140 patients with chronic pain requiring strong opioids and paracetamol ≥1.5 g/day for at least 7 days will be enrolled and randomised to either continued combination treatment or strong opioid plus placebo. In the first study phase (days 1–7), patients receive identically looking capsules containing either paracetamol at the exact dose previously used or a placebo. During a second study phase (days 7–14), all patients stop the blinded study medication (paracetamol and placebo) with follow-up to day 14. Adherence will be assessed by pill count and measurement of paracetamol and opioid serum concentrations. Patients are instructed to use a pain diary daily during the whole study. The primary outcome is the average pain score on day 7 using a 10 cm visual analogue scale (VAS). A difference between groups of ≤8 mm will be considered clinically irrelevant. Secondary outcomes will include VAS pain score on day 14, number of opioid rescue doses used, subjective ratings of overall feeling of well-being, quality of life, nausea/vomiting, drowsiness and constipation, and other adverse events, and potential effects of study drug concentrations and opioid receptor and cytochrome P450 (CYP) genotypes on the observed differences.

**Ethics and dissemination:**

The study was approved by the Ethics Committee (Ethikkommission Bern, reference number 2021-01518) and the Swiss Agency for Therapeutic Products (Swissmedic, reference number 701286). Results will be published in open-access policy peer-reviewed journals. The study is funded by the Swiss National Science Foundation (grant number 32 003B_201072).

**Trial registration number:**

NCT05088876.

**Strengths and limitations of this study**

- A randomised, double-blind, placebo-controlled, multicentre study to assess the efficacy of paracetamol when combined with strong opioids in a larger population with chronic pain.
- Inclusion of patients with and without cancer, reflecting real-world practice.
- Inclusion of genotyping of relevant opioid receptors and CYP enzymes, along with measurement of paracetamol and opioid blood concentrations.
- Recruitment challenges, such as shifts in prescription patterns or patients avoiding additional visits beyond routine care.

## Introduction

 Paracetamol (or acetaminophen) is a centrally acting analgesic and antipyretic with minimal anti-inflammatory properties. Its mechanism of action is currently not fully understood.^1^ Paracetamol is generally safe at therapeutic doses. The most important adverse effect is hepatotoxicity after supratherapeutic doses or in the presence of risk factors (eg, chronic malnutrition, alcoholism). Other possible adverse effects include chronic kidney disease,^2^ hypertension^3 4^ and peptic ulcer disease.^5^,^7^ Opioids, by contrast, act by binding to mu (µ), kappa (κ) and/or delta (δ) opioid receptors^8^ and can be classified as agonists, partial agonists or antagonists, based on their action at these receptors.^8^ Additionally, genetic differences can influence their pharmacokinetics and the response to pain treatment.^9^

The World Health Organization (WHO)’s three-step analgesic ladder, initially developed to guide treatment of patients with cancer-related pain but currently applied to chronic pain in general, provides a guide for selecting appropriate analgesics based on pain intensity. In step I, the use of a non-opioid analgesic such as paracetamol and/or a non-steroidal anti-inflammatory drug (NSAID) is recommended. If no adequate pain relief is achieved, in step II and step III of the ladder, the same step I drugs are combined with either weak (eg, tramadol) or strong (eg, morphine) opioid analgesics, respectively. Despite the widespread use of this guidance, there is currently only limited evidence showing an analgesic benefit when paracetamol is used in combination with step III opioids.

According to a systematic literature review, only insufficient evidence was found to assess the effectiveness of this combination.^10^ The review included five randomised, double-blind, controlled trials comparing strong opioids plus paracetamol versus opioid treatment alone.^11^,^15^ Limitations included the use of different opioids and daily doses across the studies, variation in the daily doses of paracetamol (between 3 and 5 g), small sample sizes (from 30 to 50 patients per study) and short duration of paracetamol use (1–7 days). Four studies^11^,^1315^ failed to find any benefit of the addition of paracetamol treatment, while one study^14^ reported improved pain control with a slight mean difference of 0.4 on a 0–10 numerical rating scale. However, this study used the highest paracetamol dose (5 g/day), which is above the recommended maximum daily dose of 4 g/day according to product characteristics, and had a short follow-up (4 days).^14^ A recent Cochrane review concluded that due to high risk of bias from the available studies, there is currently no high-quality evidence to either support or refute the use of paracetamol in combination with strong opioids for cancer pain.^16^ In this review, studies had to be randomised, ideally double-blind, include a minimum of 25 participants per treatment arm and have a duration of at least 5 days.^16^ Furthermore, pain had to be measured using a validated assessment tool such as a visual analogue scale (VAS). Based on these criteria, only three studies^11 12 15^ could be included and all were judged as having a high risk for bias due to substantial attrition (>10%) and small sample sizes. The authors concluded that the small amount and low quality of the available evidence do not indicate an additional pain-relieving effect of paracetamol in combination with strong opioids and that much larger prospective randomised studies are needed.^16^

Despite the absence of high-quality evidence and the potential of paracetamol toxicity, to date, no data from trials in a larger patient population examining the efficacy of recommended paracetamol doses added to WHO step III strong opioid treatment for chronic pain are available. In addition, no study has examined whether placebo-controlled withdrawal of the co-medication with paracetamol in patients on strong opioids negatively affects pain control. The main aim in this non-inferiority trial is to test the hypothesis that paracetamol does not provide clinically relevant benefits when given in addition to strong opioids in chronic pain patients that would justify the increased risk of paracetamol-associated adverse effects such as hepatotoxicity. This will be investigated by a blinded randomised withdrawal of fixed paracetamol co-medication. Our null hypothesis is that there is a difference between the two treatment groups, that is, strong opioids without paracetamol are inferior to strong opioids combined with paracetamol. Secondary objectives will include the investigation of the effects of drug concentrations and opioid receptor and cytochrome P450 (CYP) genotypes on VAS pain scores and differences in opioid rescue medication used, participant preference and other subjective ratings such as quality of life between arms.

## Methods and analysis

### Study setting

**Figure 1:**
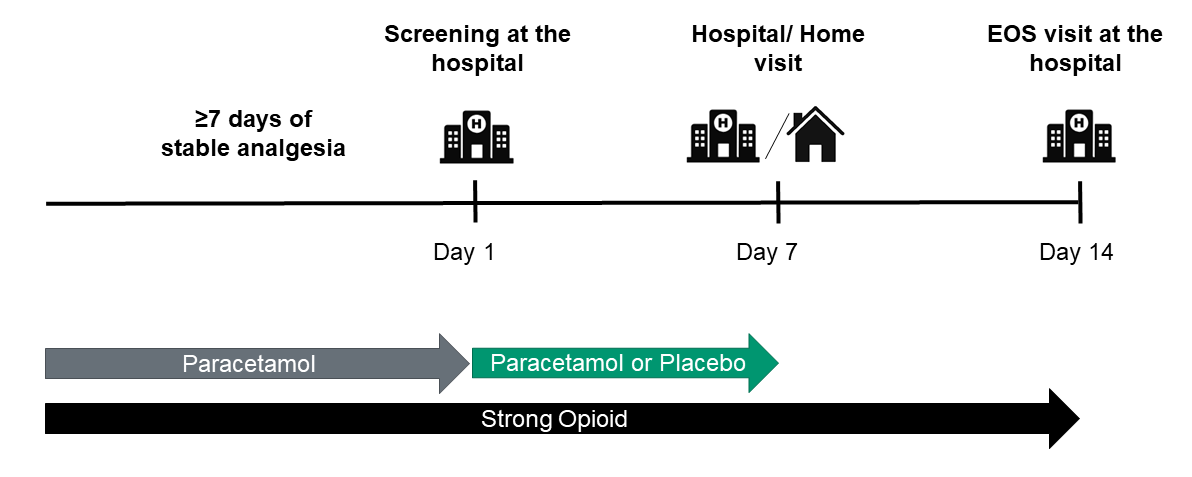
Study flow and visits. EOS, end of study.

The study will be coordinated by the central study team at the Division of Clinical Pharmacology and Toxicology, University Hospital Bern, Switzerland. Patients can be recruited from either outpatient clinics or inpatients at the University Hospital Bern and Cantonal Hospital Baden. The total study duration is 14 days, consisting of two 7-day phases. Participants will attend three hospital visits: at screening/baseline, day 7 and day 14. During these visits, blood samples will be collected, and participants will be asked about their pain levels, changes in concomitant medication and any adverse events (AEs). If mobility or transportation is an issue, the day 7 visit may be conducted as a home visit.^16^ A schematic representation of the study visits is shown in figure 1.

### Patient and public involvement

Patients and the public were not directly involved in the design and conception of the study.

### Design and study procedures

This is an investigator-initiated, randomised, double-blind, placebo-controlled, parallel-group, non-inferiority, multicentre study. Initially, the study was focused on patients with cancer. However, due to recruitment challenges and the current use of WHO guidelines in general pain management in real-life clinical settings, we decided to also include non-cancer patients with chronic pain. Hence, chronic pain patients receiving a strong opioid (ie, morphine, oxycodone, methadone, fentanyl, hydromorphone or buprenorphine) in combination with paracetamol at a minimum dose of 1.5 g/day for at least 7 days will be randomised to continue either on (blinded) paracetamol at the dose already used, or to an identically looking placebo for 7 days. The minimum dose of 1.5 g/day paracetamol was chosen since lower doses than the maximum daily dose (4 g/day) are commonly used in clinical practice (eg, Boureau *et al*^17^) and are also recommended in the Swiss product information for specific patient subgroups at a higher risk for hepatotoxicity (eg, patients with liver disease).^18^ Furthermore, because of the flat slope of the dose–response curve,^19^ only limited benefit is expected by further increasing the dose to 4 g/day, while the risk of (asymptomatic) elevation of alanine aminotransferase increases when using the maximal therapeutic dose,^20^ indicating liver damage.

Before the baseline visit, potential participants will be contacted either by phone call or directly on the wards of the hospital to review eligibility criteria (table 1).

**Table 1:** Eligibility criteria for the ParOP study

Inclusion criteria	Male and female patients receiving a WHO step III opioid (ie, morphine, oxycodone, methadone, fentanyl, hydromorphone or buprenorphine) in combination with paracetamol (minimum dose 1.5 g/day)
	Stable analgesia before randomisation, defined as no changes in the analgesic treatment during the previous 7 days
	Age ≥18 at screening
	Ability to understand the study procedures and to provide written informed consent
Exclusion criteria	Changes of the dosage or start of other (co-) analgesics (eg, tricyclic antidepressants, neuroleptics, non-steroidal anti-inflammatory drugs (NSAIDs), dipyrone), within the last 7 days preceding randomisation
	Participation in another interventional trial within 30 days prior to randomisation, with the exception of cancer treatment trials
	Surgery within the 14 days preceding randomisation or surgery planned within the duration of the study
	Any circumstances, comorbidities or conditions, which, in the opinion of the investigator, may affect full participation in the study or compliance with the study protocol

The participant information sheet and the informed consent form will be provided to interested and potentially eligible patients to have adequate time to consider trial participation and a baseline visit will be scheduled. The participant consent form in the original language (German), which is used in the study and an English translation (not part of the original study documents) is provided in online supplemental material 1. Additional strategies to expedite the recruitment process include the distribution of flyers, advertisements on the homepage of the study centres and other media, word-of-mouth advertisement and a financial compensation of 100 CHF for study participation (for travel expenses and time spent at the site during the study visits).

At the baseline visit, the eligibility criteria will be checked again, the study procedures and information will be explained (orally and in writing) and the participant will sign the informed consent form. The study team will collect the demographic data, medical and pain history (including—if applicable—cancer type and time of first diagnosis of cancer, metastases, length of chronic cancer pain treatment prior to study, other cancer treatments (eg, chemotherapy, radiotherapy), main pain diagnosis, pain localisation, pain duration/percentage time with pain per 24 hours), comorbidities, medication (including paracetamol dose, name, dose and route of strong opioid used in the 7 days prior to study begin and any potential co-analgesics/rescue medication), smoking status, risk factors for hepatotoxicity (eg, alcohol or illegal drug use), non-medical measures to relieve pain, vital signs, body weight, body height and body mass index (BMI). Additionally, questions regarding pain intensity (eg, average pain, minimum and worst pain in the last 24 hours, current pain) will be assessed by the Brief Pain Inventory (BPI)^21^ and the VAS pain score, and the quality of life with the EuroQol 5-Dimension 5-Level (EQ-5D-5L) questionnaire.^22^ Patient expectations regarding the effectiveness of paracetamol will be assessed with the expectation of treatment scale (ETS) instrument.^23^ Blood samples will be collected for laboratory tests (blood count, international normalised ratio (INR), albumin, renal and hepatic function tests) to monitor, among others, for potential development of hepatotoxicity or liver damage. Additional blood samples for genotyping of relevant CYP enzyme, mu-opioid receptor-1 (OPRM1) and catechol-O-methyltransferase (COMT) polymorphisms, as well as for concentrations of paracetamol and opioids, will be collected to explore potential influences on drug response. Any specific (nausea, vomiting, drowsiness, reduced appetite, constipation) or other AEs will be documented and their severity and classification will be assessed based on the Common Terminology Criteria for Adverse Events version 5.0 terminology.^24^ Participants will receive a pain diary along with detailed instructions on how to complete it and will be instructed to document once per day the severity of their pain using the BPI and VAS score. The pain diary also includes assessments of rescue medication used, % of time with pain in the last 24 hours, AEs and non-medical measures to relieve pain.

For the randomisation, which is expected to provide a balancing effect in the heterogeneous population by balancing known and unknown factors between the groups, participants are allocated in a 1:1 ratio to receive either paracetamol or an identically looking placebo. Block randomisation is performed using computer-generated random numbers with the clinical trial unit (CTU) Bern generating the allocation sequence. Randomisation is stratified according to the VAS pain score at baseline (VAS≤30 mm vs >30 mm) and study site. The study physicians enrol patients and assign the next sequentially coded medication pack. The allocation list is password-protected and access to the list is restricted to the database manager, the person preparing the medication packs and the personnel of the hospital pharmacy responsible in case of emergency unblinding. The ParOP study team, participants, outcome assessors and data analysts are blinded to the assignment of the patients to interventions. Unblinding will be permissible in an emergency case where knowledge of the treatment arm is necessary to provide acute medical care. In such a case, the investigator will be allowed access only to the affected participant’s allocation code.

The participants will start with the study medication (paracetamol or placebo) at the same paracetamol dose previously used directly after their screening/baseline visit and will stop the study medication on day 7. During the whole study duration, the patients are allowed to continue with any other analgesics stable prescribed prior to study inclusion. This ensures that their overall analgesic treatment remains unchanged from pre-randomisation, except substitution of paracetamol with placebo in one of the two groups after randomisation and stop of paracetamol/placebo during the second study phase. In the case of uncontrolled pain, the participants can use rescue doses of opioids. On day 7 (study visit at the hospital or at home), the participants will return the study medication bottle. The quality of life EQ-5D-5L questionnaire will be completed, concomitant medication and AEs will be assessed, blood samples will be collected for paracetamol and opioid concentrations (including the information regarding last drug intake), and the returned capsules will be counted. Measured paracetamol and opioid blood concentrations and pill counts will be used for adherence assessment. Additionally, questions will be asked regarding preference and impression of change, and study participants will be asked to guess the assigned group (placebo or paracetamol). For the last 7 days of the study, the participants in both arms will be instructed to stop the intake of paracetamol/placebo capsules. On day 14 (end of study (EOS) visit), the participants will return the pain diary to the study centre and, in addition to the assessments taking place on day 7, the vital signs and the body weight will be measured and blood will be collected for blood count, INR, albumin, renal and hepatic function tests. An overview of the study procedures is shown in table 2 and the primary and secondary study outcomes are summarised in table 3.

**Table 2:** Summary of study procedures at each study visit

	Pre-screening	Screening/baseline	Phase 1 Day 1–7 (blinded paracetamol or placebo)	Phase 2 Day 8–14 (both arms stop paracetamol/placebo)	End of study (EOS)
Visit number		1	2	3	
Time (day)	≥1 day (24 hours) before screening	1	+7	+14	
Place	Telephone	Hospital	Hospital/home	Hospital	
Eligibility check, participant information about the study	x				
Eligibility		x			
Oral and written participant information		x			
Written informed consent		x			
Randomisation to paracetamol or placebo		x			
Demographics		x			
Medical and pain history		x			
Concomitant medication		x	x	x	
Vital signs		x			x
Body weight, body height, BMI		x			x
Laboratory tests (blood count, INR, renal and hepatic function tests, albumin)		x			x
Genotyping		x			
Rating expected relief from paracetamol (ETS)		x			
Study Medication (paracetamol or placebo)			x (daily during phase 1)		
Withdrawal of study medication (paracetamol and placebo)				x (beginning of phase 2)	
VAS pain score, BPI, % time with pain/24 hours, rescue medication used, non-medical measures to relieve pain, specific adverse events (nausea, vomiting, drowsiness, appetite, constipation)		x	x (daily with pain diary)	x (daily with pain diary)	
Time of last drug intake (paracetamol, opioids)		x	x	x	
Blood concentrations paracetamol, opioids		x	x	x	
EQ-5D-5L		x	x	x	
Questions regarding preference, whether pain worse during study, and guessing of assigned group			x	x	
Adverse events		x	x	x	
Pill count			x		

BMI, body mass index; BPI, Brief Pain Inventory; EQ-5D-5L, EuroQol 5-Dimensions 5-Levels; INR, international normalised ratio; VAS, visual analogue scale.

**Table 3:** Primary and secondary outcome parameters

Outcome	Outcome measure	Time frame
Primary	VAS score (average pain intensity)	Study day 7
Secondary	Average pain using the BPI	On each study day, up to 14 days
	Minimum pain using the BPI	
	Worst pain using the BPI	
	Current pain using the BPI	
	Average pain using the BPI	During the last 4 days of each study phase (days 4–7 and 11–14)
	Minimum pain using the BPI	
	Worst pain using the BPI	
	Current pain using the BPI	
	Cumulative dose of rescue medication used	On each study day, up to 14 days and during the last 4 days of each study phase (days 4–7 and 11–14)
	Number of rescue medications used	
	Percentage increase in pain compared with baseline	During each study phase (days 1–7 and days 8–14)
	Percentage of patients meeting the predefined pain threshold	
	Subjective ratings of quality of sleep using the BPI	At baseline and during each study phase (days 1–7 and days 8–14)
	Subjective ratings of quality of life using the EQ-5D-5L questionnaire	
	Subjective ratings of functioning using the BPI and the EQ-5D-5L questionnaire	
	Patients’ expectation regarding pain relief from paracetamol prior to study treatment using the ETS	Prior to study treatment
	Question about participants’ preference (study vs baseline)	At day 7 and 14
	Participants’ impression of change	
	Participants’ guess regarding their assigned group (verum or placebo)	
	Assessment of serious adverse events	During each study phase (days 1–7 and days 8–14)
	Assessment of specific adverse events: nausea/vomiting, appetite, constipation, drowsiness	
	Assessment of other adverse events	
	Number of withdrawals from study or treatment	
	Time (days) of stable pain control	During whole study (days 1–14)
	Patients’ potential to develop hepatotoxicity (investigation of risk factors)	
	% hours with pain/24 hours	On each study day, up to 14 days

BPI, Brief Pain Inventory; EQ-5D-5L, EuroQol 5-Dimensions 5-Levels; ETS, expectation of treatment scale; VAS, visual analogue scale.

### Estimated sample size and power

The sample size is based on the primary outcome of VAS pain at day 7 to demonstrate non-inferiority of opioid treatment only vs the combination of opioids and paracetamol. The acceptable non-inferiority margin for pain was assessed based on clinical experience, empirical evidence from patients with cancer^25^ and previous reports evaluating analgesic effects.^26 27^ A difference of 8 mm or less on a 100 mm VAS pain score will be considered not clinically relevant. Findings of previous studies showed an SD of approximately 15 mm regarding changes on VAS pain scores.^11^ Assuming equal means and the same SD of 15 mm in the two groups, a power of 0.8 and an alpha error of 0.025 (one-sided; to be consistent with a two-sided 0.05 test), a non-inferiority margin of 8 mm on the VAS score for pain requires 112 participants. To account for a 20% dropout rate, we intend to recruit 140 patients. Secondary outcomes and subgroup analyses will be investigated in an exploratory manner and no further sample size or power calculations have been performed for these analyses.

### Data management

Data will be recorded in electronic case report forms using REDCap (Research Electronic Data Capture) and paper-based worksheets, where necessary. The electronic database REDCap is maintained by CTU Bern, with access restricted to trained study personnel. Each data entry is time-stamped and linked to the responsible user, with all retrospective changes logged in an audit trail. Regular central data monitoring will be conducted by the study team. Additionally, a qualified monitor from CTU Bern, independent from the study, will perform quality control checks of both study conduct and data integrity (one monitoring visit per site when at least 8–10 participants have completed the study). The Sponsor-Investigator will keep the trial master file, the extracted data, the metadata and interim/final reports for at least 20 years following study completion or early termination.

### Exploratory variables

Genetic polymorphisms of CYP enzymes can result in differences in drug concentrations and patient response and thus influence opioid dosing and prescription practices.^9^ For example, for the polymorphic CYP2D6, ‘poor’ and ‘ultra-rapid’ metabolisers have been described, based on the presence of two non-functional (null alleles, little or no enzymatic activity) or more than two functional alleles (gene duplication), respectively,^9^ while the CYP2B6*6 genetic variation has been reported to play a potential role in the response to methadone maintenance therapy.^9 28^

Further genetic variations of interest regarding pain management and analgesic response to opioids are the OPRM1 and the COMT genes. The OPRM1 gene encodes the mu-opioid receptors and presents a commonly investigated single-nucleotide polymorphism with a transition from A to G on nucleotide 118 (A118G).^29^ In previous studies, carriers of the 118G OPRM1 variant required higher morphine doses,^30^ experienced less postoperative nausea and vomiting,^31^ and showed a reduction of mu-opioid receptor availability and lower placebo-induced mu-opioid system activation^29^ than homozygous carriers of 118A, thus indicating that this polymorphism might be of relevance for pain management and treatment responses. The COMT is a pivotal enzyme of catecholamine metabolism^32^ with the common functional polymorphism Val158Met. In previous studies, individuals homozygous for the Met158 (low activity) allele showed decreased responses to pain compared with heterozygotes^33^ and patients with cancer homozygous for the Val158 (high activity) allele needed more morphine than homozygous Met158 patients with cancer.^34^ Therefore, blood samples for genotyping and measuring the opioid drug concentrations will be performed in order to investigate potential genetic influences on drug response and adverse effects.

### Statistical analysis

The statistical analysis (intention-to-treat (ITT) and per protocol analyses (PP)) will be performed by a blinded statistician. For the primary outcome, both analyses should reach the same conclusion to establish non-inferiority. For the remaining outcomes, the ITT analysis will be performed for the primary analysis and the PP approach for the secondary analysis. The primary and secondary pain outcomes will be analysed using a linear repeated measures mixed model. Participants will be treated as a random effect, with fixed effects for day, treatment group, their interaction and baseline VAS. Non-inferiority of the primary outcome will be based on the CI of the difference in the VAS score between the two groups at day 7. If the upper limit of the one-sided 97.5% CI is below the non-inferiority margin of 8 mm, opioids alone will be considered non-inferior to opioids combined with paracetamol.

Other continuous outcomes will also be evaluated in linear, repeated-measures, mixed-effects models. The assessment of the use of rescue opioid medication each day will be evaluated by a logistic mixed-effects model, while the comparison between the two groups regarding the cumulative dose/number of rescue medication will be analysed with a generalised linear model. AE and serious adverse events (SAE) as well as the number of withdrawals will be presented in each group using proportions with associated 95% CIs. For the identification of clinical predictors of stable pain control, a multivariable logistic model will be used with the following variables: sex, age, patient’s expectation, specific opioids and co-analgesics used, drug concentrations and doses, genotype, baseline VAS, cancer type and length of diagnosis, pain localisation and quality, and length of pain treatment.

The influence of covariates such as demographics, cancer type, medication use or genotypes on time-to-event (TTE) outcomes will be analysed using Kaplan-Meier curves, log-rank tests and TTE models as supported by the data.^35 36^ A TTE model of stable pain control in the study population would allow for exploring different medication schedules to optimise pain control. As similar trials have suffered from attrition bias, we will consider the impact of informative drop-outs. Reasons for dropping out include adverse reactions, changes in health status or lack of treatment effectiveness. In these cases, drop-outs should be considered informative as opposed to random or completely random drop-outs. The longitudinal outcome of VAS scores in both groups and the drop-out process can be analysed using risk-regression models or cause-specific Cox proportional hazards models, if attrition rates are high enough to warrant such a procedure. Survival data of time to first pain events will be analysed by log-rank tests. TTE analysis will consider exponential, Weibull, Gompertz, gamma and generalised gamma models.

Repeated-measures, mixed-effects models partly account for missing data as long as at least one outcome assessment is available. If, nevertheless, more than 10% of patients have completely missing outcome data, we will employ multiple imputation.

### Clinical implications

Chronic pain remains a major public health problem, with a global prevalence estimated at 28%, leading to additional health challenges and a reduced quality of life.^37^ Patients with cancer often suffer from pain, with one-third reporting it as moderate to severe.^38^ Insurance claims data indicate that over 50% of patients prescribed strong opioids also received paracetamol.^39^ Although paracetamol is considered safe, even recommended doses can cause elevations in liver enzymes in healthy individuals,^20^ whereas the risk of hepatotoxicity increases in the presence of risk factors (eg, CYP-inducers, chronic malnutrition, regular intake of alcohol).^40 41^ If our hypothesis that paracetamol offers no additional clinical benefit when combined with strong opioids proves correct, unnecessary exposure of patients to the risk of paracetamol-associated adverse effects could be avoided. Furthermore, each additionally prescribed medication increases the risk of drug–drug interactions and adverse drug reactions, which can lead to hospital admissions. Polypharmacy not only reduces quality of life, especially when medications offer limited benefit, but also increases the risk of non-adherence.^42^ Thus, removing unnecessary paracetamol prescriptions and reducing the pill burden could meaningfully improve patient safety and outcomes.

If, contrary to our initial hypothesis, paracetamol improves analgesia when combined with a strong opioid, the findings would be highly important as well. Opioid-sparing agents are urgently needed to reduce the risk of opioid dependence in pain patients. Monitoring the daily need for opioid rescue medications in both groups will offer valuable insights into the potential opioid sparing effect of paracetamol. If the patients in the placebo group require higher opioid doses, this would highlight paracetamol’s role as an effective adjunct therapy. This is particularly important given the ongoing opioid crisis,^43^ driven by increased opioid prescribing and associated with SAEs and mortality.^44^,^47^ Finally, the assessment of genotyping alongside opioid concentration measurements will enable an exploration of genetic influences on drug response and adverse effects, contributing to more personalised pain management strategies in the future.

## Ethics and dissemination

The study is conducted in accordance with the Declaration of Helsinki, the guidelines of Good Clinical Practice issued by the International Conference on Harmonisation, the Swiss Law and the Swiss regulatory authority’s requirements. The study is approved by the Ethics Committee (Ethikkommission Bern, reference number 2021-01518) and the Swiss Agency for Therapeutic Products (Swissmedic, reference number 701286) and registered in the Clinical Trials Registry Platform of the National Institute of Health (NIH), ClinicalTrials.gov (NCT05088876) and the Swiss National Clinical Trials Portal (SNCTP). Protocol amendments will be communicated with and approved by the Ethics Committee and Swissmedic before implementing them in the study. The study procedures will be explained to participants both orally and in writing. The study team will answer all questions and inform the participants that they may withdraw from the study at any time without any impact on their further medical treatment. All candidates will be given at least 24 hours to consider participation and will provide written informed consent voluntarily. Confidentiality will be ensured through the use of unique identification code numbers.

Study participants who develop a contraindication to continuing the intervention (eg, an AE/SAE, unexpected pain exacerbation with no response to rescue medication, or administrative reasons) may prematurely discontinue the trial treatment. Participants who would like to withdraw from the study will be encouraged to attend a discontinuation/EOS visit. The Sponsor-Investigator will be responsible for terminating the study early in case of insufficient participant recruitment, safety concerns, early or external evidence of benefit or harm of the intervention or alterations in clinical practices. Insurance coverage will be provided for the study participants by the sponsor-investigator’s institute. The study group is an independent academic research group, which will not employ professional writers and comply with the open access regulation of the Swiss National Science Foundation. The full study protocol, statistical code and informed consent materials will be available on request. We plan to register and deposit data related to individual, primary publications rather than the entire study database in the Bern Open Repository and Information System.

## Supplementary material

10.1136/bmjopen-2025-107360 online supplemental file 1
