# Supplementary material for: Efficacy of paracetamol added to WHO step III opioids in chronic pain control: study protocol for a randomised, double-blind, placebo-controlled, non-inferiority, multicentre study in Switzerland
Source: BMJ Open. 2025 Dec 31;15(12):e107360. doi: 10.1136/bmjopen-2025-107360 (PMC12766828; doi:10.1136/bmjopen-2025-107360)
Supplement: online supplemental file 1 [file bmjopen-15-12-s001.docx]

**
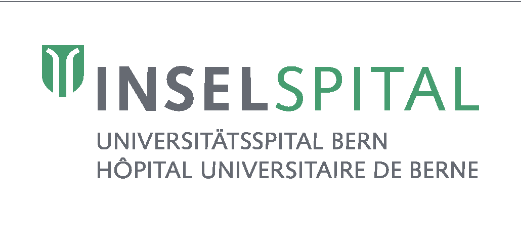
**

**Effect of paracetamol in combination with strong opioids according to WHO recommendations in chronic cancer pain**

| Original title of the study: | “Paracetamol in addition to WHO Step III opioids in chronic cancer pain control – a randomized, double-blind, placebo-controlled, non-inferiority study” |
| --- | --- |
| The study is organized by: | Inselspital Bern |

Dear Madam, Dear Sir,

We would like to ask you if you are willing to participate in a clinical study. The study is described below:

1. **Aim of the study**

The aim of this study is to investigate whether omitting paracetamol, according to WHO recommendations, in combination with strong opioids has an effect on pain perception. The data obtained will help optimize guidelines for pain treatment, since potential adverse effects of therapy with paracetamol could either be avoided (if no benefit of the combination is shown) or better justified (if paracetamol proves to be more effective than placebo).

1. **Selection**

Patients aged 18 years or older who are already receiving paracetamol and a strong opioid may participate. Persons whose pain therapy has had to be adjusted in the last 7 days or who are scheduled for surgery in the coming days are excluded. After surgery, patients must wait at least 14 days before participating.

1. **General information about the study**

Paracetamol is approved for the treatment of mild to moderate pain. When used correctly, it rarely causes side effects, but in cases of overdose or under certain risk factors, it can lead to liver failure. Elevated liver enzyme levels may also occur in some healthy individuals, even with normal dosages. Based on WHO recommendations for pain treatment, paracetamol is often given together with strong opioids, despite lacking clear evidence for the benefit of this combination.

In the study, 140 patients who are already receiving paracetamol and a strong opioid will be randomly assigned to two groups: one group will continue to receive paracetamol, the other group will instead receive a placebo for 7 days, which looks identical to paracetamol but has no active ingredient. The likelihood of assignment to either group is equal. The study is conducted double-blind (neither the participants nor the study team know the group assignment). In a second phase, both groups will discontinue paracetamol or placebo for 7 days.

The study will be conducted at Inselspital / University Hospital Bern, according to applicable Swiss law, internationally recognized principles and with approval from the responsible ethics committee and the Swiss drug authority, Swissmedic. The entire study lasts 14 days from the initial examination to the final visit. A description of the study is also available on the Federal Office of Public Health’s website: www.kofam.ch

1. **Procedure**

Interested individuals contact us and first have a phone conversation with a study team member to assess potential eligibility and, if appropriate, schedule a preliminary examination.

At the preliminary exam, it will be determined whether you are suitable for participation. Demographic data (age, sex, education, etc.), medical conditions, medications, vital signs (heart rate, blood pressure), body weight, and height will be collected. Your medical history and medication use will also be reviewed via the electronic clinical information system of Inselspital. You will be asked about pain and well-being. Blood samples will be taken to assess blood count, albumin, coagulation, liver and kidney function, paracetamol and opioid concentrations, and—with your specific consent—genes relevant to opioid metabolism and effect. No more than 100 ml of blood will be taken over the entire study (for comparison: a blood donation is about 450 ml).

After the preliminary exam, participants are randomly assigned to one of the two groups in a double-blind fashion: one group will continue paracetamol, the other will receive placebo for 7 days. In a second phase, both groups will discontinue paracetamol/placebo for 7 days. Participants should keep a pain diary, documenting daily their pain and medication use, including any rescue pain medication if needed.

On days 7 and 14, participants will return to Inselspital. At these visits, questions will be asked regarding well-being, pain, side effects, and medication intake; blood levels of paracetamol and opioids will also be measured. Participants will also be asked to guess which group they were assigned to.

On day 14, the final visit takes place. Blood tests will again be performed for the same parameters as at baseline.

It may be necessary to withdraw you from the study early, for example, due to side effects or intolerances.

Your physician will be informed about your study participation, which will also be documented in the electronic medical records.

The figure shows an overview of all appointments during the study:


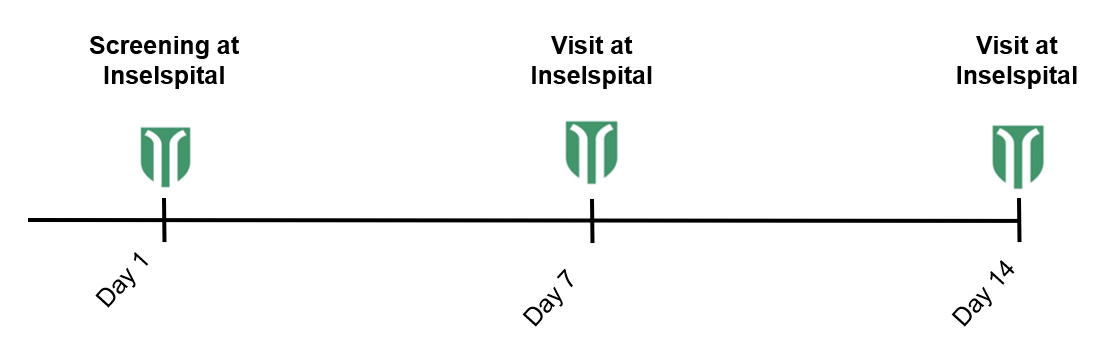


1. **Benefits**

By participating, you contribute to improving guidelines for pain treatment. The data obtained will support future pain management research. There is no direct medical benefit for participants.

1. **Rights**

Participation is voluntary. If you decline or withdraw later, you do not need to give a reason. Your medical care will not be affected. You may ask questions at any time. Contact persons are listed at the end of this information.

1. **Obligations**

As a participant, you are obliged to:

- Follow the medical instructions of the study team and comply with the study plan.
- Report truthfully about your pain, new symptoms, complaints, or changes in well-being.
- Inform the study physician about treatment by another doctor or use of any medications (including complementary medicine).
- Women of childbearing potential: inform the study physician if you become pregnant.

1. **Risks**

Blood sampling may rarely cause small bruises at the puncture site, which usually heal within a few days. Very rarely, inflammation may occur at the site; if this happens, please notify the study doctor immediately.

**Women of childbearing potential**

If you become pregnant during the study, you must inform the study physician immediately. You will be asked for information about the course and outcome of the pregnancy, and the physician will discuss further steps with you.

1. **Results**

The study team will inform you during the study of any new findings that may affect the benefit or safety of participation. You will receive this information orally and in writing.

If incidental findings relevant for prevention, detection, or treatment of diseases are discovered, you will be informed, and—if you wish—your treating physician will also be notified.

1. **Data and Samples confidentiality**

Your personal and medical data will be collected. Only a few authorized professionals will see your unencrypted data, strictly to carry out study tasks. For research purposes, data will be encrypted, meaning that identifying information (name, date of birth) is replaced by a code. Those without access to the key cannot link data to you. Publications will contain only aggregated, anonymous data. Your name will never appear online or in publications.

If raw data are required by a journal for publication, they will still be coded. The key list always remains at the hospital. All personnel are bound by confidentiality and data protection rules. You may access your data at any time.

Authorities (e.g. ethics committee, Swissmedic) may review the study, in which case your data may be disclosed. Insurance companies may also need access in case of damage claims. All parties are bound to confidentiality.

1. **Withdrawal**

You may withdraw at any time. Data and samples collected up to that point will still be analyzed in coded form to preserve the value of the study. After analysis, data and samples will be stored securely as required by law and destroyed after publication of results. Please consider this before deciding to participate.

1. **Compensation**

Neither you nor your health insurance will incur costs. All study-related procedures are free. Participants will receive financial compensation of 100 CHF (for travel expenses and time).

1. **Liability**

Die Institution (der Sponsor), die die Studie veranlasst hat und für die Durchführung verantwortlich ist, haftet für Schäden, welche Ihnen im Zusammenhang mit der getesteten Substanz oder Forschungshandlungen (z.B. Untersuchungen) entstehen könnten. Die Voraussetzungen und das Vorgehen dazu sind gesetzlich geregelt. Das Inselspital hat daher eine Versicherung (Zürich Versicherungsgesellschaft) abgeschlossen, um in einem möglichen Schadenfall für die Haftung aufkommen zu können. Falls Sie einen Schaden erlitten haben, so wenden Sie sich bitte an den/die Prüfarzt/-ärztin.

1. **Funding**

The study is funded by the Swiss National Science Foundation.

1. **Contact person**

For questions, concerns, or emergencies during or after the study, please contact:

- Prof. Dr. med. E. Liakoni, Tel. 031 632 54 61, E-Mail [evangelia.liakoni@insel.ch](mailto:evangelia.liakoni@insel.ch)
- Emergency related to the study: 077 406 81 96
- On-call physician in Clinical Pharmacology: 031 632 21 11 (Monday - Friday 08:00- 17:00, in emergencies outside these hours, until 20:00 week days and 18: 00 weekends/ holidays)

In a life-threatening emergency, call 144 or go to the nearest emergency department.

**Declaration of concent**

**Written consent to participate in a research project**

Please read this form carefully. Ask if there is anything you do not understand. Your written consent is required for participation.

| **BASEC-Nummer:** | 2021-01518 |
| --- | --- |
| **Title oft he study** | Paracetamol in addition to WHO Step III opioids in chronic cancer pain control – a randomized, double-blind, placebo-controlled, non-inferiority study |
| **Responsible institution (Sponsor with Adresse**): | Inselspital, Universitätsspital Bern, Freiburgstrasse 8, 3010 Bern |
| **Study site**: | Inselspital, Bern |
| **Principle investigator:** First and last name: | Prof. Dr. med. Evangelia Liakoni |
| **Participant:**  First and last name: Date of birth: | female  male |

- I have been informed verbally and in writing by the undersigned investigator about the purpose, procedure, possible advantages and disadvantages, and potential risks of the study.
- I participate voluntarily and accept the content of the written information provided. I had sufficient time to make my decision.
- My questions have been answered. I keep the written information and receive a copy of this consent form.
- I agree that authorized staff of the sponsor and ethics committee may inspect my unencrypted data for monitoring, while confidentiality is strictly maintained.
- I will be informed of study results that directly affect my health.
- I can withdraw at any time without giving reasons. My medical care remains guaranteed. Data and samples collected until withdrawal will still be used for study analysis.
- I am informed that insurance covers any study-related harm.
- I am aware of my obligations stated in the study information. For my safety, the investigator may exclude me from the study at any time.

| Place, Date | Participant’s signature |
| --- | --- |

**Investigator’s confirmation:** I confirm that I have explained the nature, significance, and scope of the study to this participant. I assure compliance with all legal requirements and obligations related to this study. If at any time I learn of aspects that may affect the participant’s willingness to continue, I will immediately inform them.

| Place, Date | Name and Last name of investigator  Investigator’s signature |
| --- | --- |

**
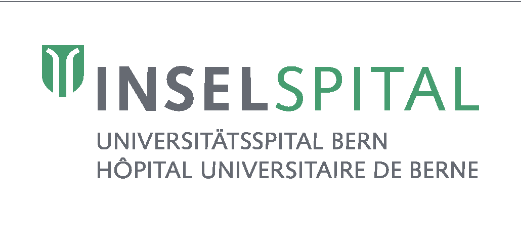
**

**Effekt von Paracetamol in der Kombination mit starken Opioiden nach Empfehlung der WHO bei chronischen Tumorschmerzen**

| Original Titel der Studie: | “Paracetamol in addition to WHO Step III opioids in chronic cancer pain control – a randomized, double-blind, placebo-controlled, non-inferiority study” |
| --- | --- |
| Die Studie ist organisiert durch: | Inselspital Bern |

Sehr geehrte Dame, sehr geehrter Herr,

Wir möchten Sie anfragen, ob Sie an einer klinischen Studie teilnehmen wollen. Im Folgenden wird Ihnen dieses Studienvorhaben dargestellt:

1. **Ziel der Studie**

Ziel dieser Studie ist zu untersuchen, ob sich das Weglassen von Paracetamol nach Empfehlungen der WHO in Kombination mit starken Opioiden auf das Schmerzempfinden auswirkt. Die gewonnenen Daten werden helfen, die Richtlinien bei Schmerzen zu optimieren, da die potentiellen unerwünschten Wirkungen bei einer Therapie mit Paracetamol entweder vermieden (wenn kein Nutzen durch die Kombination gezeigt wird), oder besser gerechtfertigt werden könnten (falls Paracetamol sich als wirksamer als Placebo erweist).

1. **Auswahl**

An der Studie können Patientinnen und Patienten teilnehmen, welche 18 Jahre alt oder älter sind und bereits Paracetamol und ein starkes Opioid erhalten. Personen, bei denen die Schmerztherapie in den letzten 7 Tagen angepasst werden musste oder bei denen eine Operation in den nächsten Tagen geplant ist, sind von der Studienteilnahme ausgeschlossen. Nach einer Operation müssen Patienten und Patientinnen mindestens 14 Tage warten, um an der Studie teilnehmen zu können.

1. **Allgemeine Informationen zur Studie**

Paracetamol ist für die Behandlung von milden bis mässigen Schmerzen zugelassen. Bei normalem Gebrauch führt Paracetamol selten zu unerwünschten Wirkungen, es kann jedoch zum Leberversagen bei Überdosierung oder bestimmten Risikofaktoren kommen. Ausserdem zeigt sich eine Erhöhung der Leberwerte im Blut auch unter der normalen Dosierung bei manchen gesunden Personen. Basierend auf Empfehlungen der WHO zur Schmerzbehandlung wird Paracetamol oft zusammen mit starken Opioiden gegeben, trotz fehlender Daten zu einem klaren Nutzen dieser Kombination.

Im Rahmen der Studie werden 140 Patientinnen und Patienten, welche bereits Paracetamol und ein starkes Opioid erhalten, gemäss Zufallsprinzip in zwei Gruppen eingeteilt: Eine Gruppe wird weiterhin Paracetamol erhalten, die zweite Gruppe wird stattdessen 7 Tage lang ein Placebo erhalten, welches genau wie das Paracetamol aussieht, aber keinen Wirkstoff hat.

Die Wahrscheinlichkeit in eine der zwei Gruppen zugeteilt zu werden ist gleich hoch. Die Studie wird doppelblind durchgeführt (weder die Teilnehmenden noch das Studienteam wissen, welcher Gruppe sie zugeteilt wurden). In einer zweiten Phase wird das Paracetamol bzw. das Placebo in beiden Gruppen 7 Tage lang abgesetzt.

Die Studie wird am Inselspital / Universitätsspital Bern nach geltenden schweizerischen Gesetzen und nach international anerkannten Grundsätzen durchgeführt, sowie nach Genehmigung durch die zuständige Ethikkommission und die Schweizerische Arzneimittelbehörde Swissmedic. Die gesamte Studie dauert von der Eintrittsuntersuchung bis zum Abschluss 14 Tage. Eine Beschreibung dieser Studie finden Sie auch auf der Internetseite des Bundesamtes für Gesundheit: [www.kofam.ch](http://www.kofam.ch).

1. **Ablauf**

Interessierte nehmen mit uns Kontakt auf und führen dann zunächst ein Telefonat mit einer Person des Studienteams, um festzustellen, ob eine Studienteilnahme grundsätzlich in Frage kommt und wenn ja, um einen Termin für eine Voruntersuchung zu vereinbaren.

An der Voruntersuchung wird genauer geprüft, ob Sie für die Teilnahme an dieser Studie geeignet sind. Im Rahmen der Studie werden demographische Daten wie Alter, Geschlecht, Bildungsgrad etc., Erkrankungen, eingenommene Medikamente, Vitalzeichen wie Herzfrequenz und Blutdruck, sowie Körpergewicht und –grösse erfasst. Für die vollständige Erfassung Ihrer aktuellen medizinischen Daten (Erkrankungen, eingenommene Medikamente etc.) wird auf das elektronische Klinikinformationssystem des Inselspitals zurückgegriffen. Es werden Fragen hinsichtlich Schmerzen und Wohlempfinden gestellt. Blutproben zur Bestimmung des Blutbildes, des Albumins, der Gerinnung, der Leber- und Nierenfunktion, der Konzentrationen von Paracetamol und Opioiden, sowie nach spezieller Einwilligung auch zur Bestimmung der für Opioidstoffwechsel und –wirkung relevanten Gene, werden abgenommen. Maximal werden Ihnen nicht mehr als 100 ml Blut während der gesamten Studie abgenommen (bei einer Blutspende sind es etwa 450 ml).

Im Anschluss an die Voruntersuchung werden die Studienteilnehmenden doppelblind gemäss Zufallsprinzip in die zwei Gruppen eingeteilt: Eine Gruppe wird weiterhin Paracetamol, die zweite Gruppe wird stattdessen 7 Tage lang ein Placebo erhalten. In einer zweiten Phase wird das Paracetamol bzw. das Placebo in beiden Gruppen für 7 Tage abgesetzt. Die Studienteilnehmenden sollten während der gesamten Studie Ihre Schmerzen wie auch die eingenommenen Schmerzmittel mittels eines von der Studie zur Verfügung gestellten Schmerztagebuches täglich dokumentieren. Im Fall von Schmerzen während beiden Studienphasen kann die Einnahme einer Schmerzreservemedikation erfolgen, diese ist ebenfalls in dem Schmerztagebuch zu dokumentieren.

Sieben und 14 Tage nach Beginn der Studie (Ende von erster und zweiter Phase) findet ein Termin am Inselspital statt. Bei diesen Terminen werden Fragen zum Wohlempfinden und Schmerzen, unerwünschten Wirkungen und Einnahme von Medikamenten gestellt und die Blutkonzentrationen von Paracetamol und Opioiden werden gemessen. Studienteilnehmende werden dann auch gebeten zu erraten, in welche Gruppe sie eingeteilt wurden.

Am Tag 14 findet die Abschlussuntersuchung statt und die Teilnehmenden werden von der Studie entlassen. Beim Termin werden Blutproben zur Bestimmung der selben Laborparameter wie am Studienbeginn abgenommen.

Es kann sein, dass wir Sie von der Studie vorzeitig ausschliessen müssen. Das kann z.B. wegen unerwünschter Wirkungen oder Unverträglichkeitsreaktionen geschehen.

Ihr/e Arzt/Ärztin wird über die Studienteilnahme informiert, diese wird ebenfalls im elektronischen Klinikinformationssystem dokumentiert.

Die Abbildung zeigt eine Übersicht über alle Termine während der Studie:

**
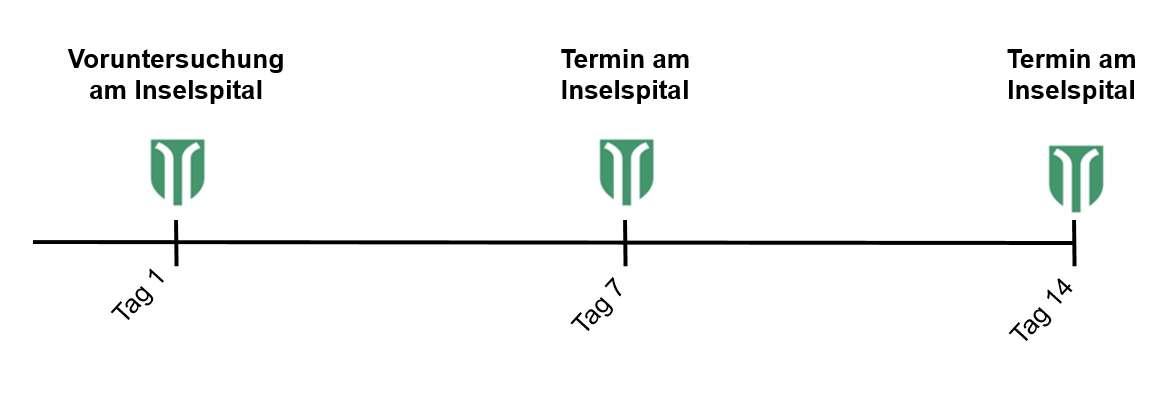
**

1. **Nutzen**

Durch die Teilnahme an dieser Studie tragen Sie dazu bei, die Richtlinien bei Schmerzen zu optimieren. Die gewonnenen Daten sind ein Beitrag an die Forschung und dienen dazu, Patientinnen und Patienten künftig bei der Schmerzbehandlung gezielter zu unterstützen. Für die Studienteilnehmenden ergibt sich kein medizinischer Nutzen.

1. **Rechte**

Sie nehmen an der Studie freiwillig teil. Wenn Sie nicht mitmachen oder später Ihre Teilnahme zurückziehen wollen, müssen Sie dies nicht begründen. Ihre medizinische Behandlung/Betreuung ist unabhängig von Ihrem Entscheid gewährleistet. Sie dürfen jederzeit Fragen zur Studienteilnahme stellen. Wenden Sie sich dazu bitte an die Personen, die am Ende dieser Information genannt sind.

1. **Pflichten**

Als Studienteilnehmende sind Sie verpflichtet:

- Den medizinischen Anweisungen des Studienteams zu folgen und sich an den Studienplan zu halten
- Wahrheitsgetreu über den Verlauf der Schmerzen, neue Symptome, neue Beschwerden oder Änderungen im Befinden zu informieren
- Den/die Prüfarzt/-ärztin über die gleichzeitige Behandlung bei einem/einer anderen Arzt/Ärztin und über die Einnahme von Medikamenten (auch der Komplementärmedizin) zu informieren
- Frauen im gebärfähigen Alter: Den/die Prüfarzt/-ärztin im Fall einer Schwangerschaft zu informieren

1. **Risiken**

Bei der Blutentnahme kann es selten zu kleinen Blutergüssen (Hämatomen) um die Einstichstelle kommen, die jedoch in wenigen Tagen abheilen. In sehr seltenen Fällen kann es zu einer Entzündung der Einstichstelle kommen. Wenn dies der Fall sein sollte, bitten wir Sie, dies umgehend dem Studienarzt/der Studienärztin zu melden.

**Frauen im gebärfähigen Alter**

Sollten Sie während der Studie schwanger werden, müssen Sie Ihre/-n Prüfarzt/-ärztin sofort informieren. In diesem Fall werden Sie gebeten, Angaben über den Verlauf und den Ausgang der Schwangerschaft zu machen. Der/die Prüfarzt/-ärztin wird mit Ihnen das weitere Vorgehen besprechen.

1. **Ergebnisse**

Das Studienteam wird Sie während der Studie über alle neuen Erkenntnisse informieren, die den Nutzen der Studie oder Ihre Sicherheit und somit Ihre Einwilligung zur Teilnahme an der Studie beeinflussen können. Sie werden die Information mündlich und schriftlich erhalten.

Bei Zufallsbefunden, die bei Ihnen zur Verhinderung, Feststellung und Behandlung bestehender oder künftig zu erwartenden Krankheiten beitragen können, werden Sie informiert und sofern Sie das wünschen, werden diese Befunde auch Ihrem/Ihrer behandelnden Arzt/Ärztin mitgeteilt.

1. **Vertraulichkeit von Daten und Proben**

Für diese Studie werden Ihre persönlichen und medizinischen Daten erfasst. Entscheiden Sie sich für eine Studienteilnahme, werden nur sehr wenige Fachpersonen Ihre unverschlüsselten Daten sehen und zwar ausschliesslich, um Aufgaben im Rahmen der Studie zu erfüllen. Bei der Datenerhebung zu Studienzwecken werden die Daten verschlüsselt. Verschlüsselung bedeutet, dass alle Bezugsdaten, die Sie identifizieren könnten (Name, Geburtsdatum), gelöscht und durch einen Schlüssel ersetzt werden. Diejenigen Personen, die den Schlüssel nicht kennen, können daher keine Rückschlüsse auf Ihre Person ziehen. Bei einer Publikation sind die zusammengefassten Daten daher auch nicht auf Sie als Einzelperson rückverfolgbar. Ihr Name taucht niemals im Internet oder einer Publikation auf. Manchmal gibt es die Vorgabe bei einer Zeitschrift zur Publikation, dass Einzel-Daten (sogenannte Roh-Daten) übermittelt werden müssen. Wenn Einzel-Daten übermittelt werden müssen, dann sind die Daten immer verschlüsselt und somit ebenfalls nicht zu Ihnen als Person rückverfolgbar. Die Schlüssel-Liste bleibt immer in der Institution/dem Spital. Alle Personen, die Einsicht in Ihre Daten haben, unterstehen der Schweigepflicht. Alle Vorgaben des Datenschutzes werden eingehalten. Sie als teilnehmende Person haben jederzeit das Recht auf Einsicht in Ihre Daten.

Möglicherweise wird diese Studie durch zuständige Behörden (z.B. Ethikkommission, Swissmedic) überprüft. In diesem Zusammenhang müssen eventuell Ihre persönlichen und medizinischen Daten offengelegt werden. Ebenso kann es sein, dass bei Schäden ausnahmsweise eine Versicherung die Daten der Studienteilnehmenden einsehen muss. Alle Personen müssen absolute Vertraulichkeit wahren.

1. **Rücktritt**

Sie können jederzeit aufhören und von der Studie zurücktreten, wenn Sie das wünschen. Die bis dahin erhobenen Daten und Proben werden noch verschlüsselt ausgewertet, weil das ganze Projekt sonst seinen Wert verliert. Nach der Auswertung werden die Daten und die Proben weiterhin verschlüsselt nach den gesetzlichen Vorgaben aufbewahrt. Nach der finalen Publikation der Resultate werden die Proben vernichtet. Prüfen Sie bitte, ob Sie damit einverstanden sind, bevor Sie bei der Studie mitmachen.

1. **Entschädigung**

Es entstehen Ihnen oder Ihrer Krankenkasse keine Kosten durch die Teilnahme. Sämtliche Untersuchungen, die im Rahmen der Studie erfolgen, sind kostenlos. Teilnehmende erhalten eine finanzielle Entschädigung von 100 CHF für die Studienteilnahme (Reisekosten und Zeitaufwand während der Studienvisiten).

1. **Haftung**

Die Institution (der Sponsor), die die Studie veranlasst hat und für die Durchführung verantwortlich ist, haftet für Schäden, welche Ihnen im Zusammenhang mit der getesteten Substanz oder Forschungshandlungen (z.B. Untersuchungen) entstehen könnten. Die

Voraussetzungen und das Vorgehen dazu sind gesetzlich geregelt. Das Inselspital hat daher eine Versicherung (Zürich Versicherungsgesellschaft) abgeschlossen, um in einem möglichen Schadenfall für die Haftung aufkommen zu können. Falls Sie einen Schaden erlitten haben, so wenden Sie sich bitte an den/die Prüfarzt/-ärztin.

1. **Finanzierung**

Die Studie wird durch den Schweizerischen Nationalfonds finanziert.

1. **Kontaktperson**

Bei Fragen, Unsicherheiten oder Notfällen, die während der Studie oder danach auftreten, können Sie sich jederzeit an eine dieser Kontaktpersonen wenden:

- Prof. Dr. med. E. Liakoni, Tel. 031 632 54 61, E-Mail [evangelia.liakoni@insel.ch](mailto:evangelia.liakoni@insel.ch)
- Bei Notfällen im Zusammenhang mit der Studie: 077 406 81 96
- Dienstarzt/Dienstärztin klinische Pharmakologie via Zentrale des Universitätsspitals Bern: 031 632 21 11 (Montag bis Freitag von 08:00 Uhr bis 17:00 Uhr, bei Notfällen ausserhalb der Präsenzzeiten kann der Hintergrunddienst werktags bis 20:00 Uhr und an Wochenenden und Feiertagen bis 18:00 Uhr kontaktiert werden)

In einem lebensbedrohlichen Notfall, rufen Sie die Nummer 144 an oder suchen Sie die Notfallstation des nächstgelegenen Spitals auf.

**Einwilligungserklärung**

**Schriftliche Einwilligungserklärung zur Teilnahme an einem Studienprojekt**

Bitte lesen Sie dieses Formular sorgfältig durch. Bitte fragen Sie, wenn Sie etwas nicht verstehen oder wissen möchten. Für die Teilnahme ist Ihre schriftliche Einwilligung notwendig.

| **BASEC-Nummer:** | 2021-01518 |
| --- | --- |
| **Titel der Studie (wissenschaftlich und Laiensprache)**: | Paracetamol in addition to WHO Step III opioids in chronic cancer pain control – a randomized, double-blind, placebo-controlled, non-inferiority study / Effekt von Paracetamol in der Kombination mit starken Opioiden nach Empfehlung der WHO bei chronischen Tumorschmerzen |
| **verantwortliche Institution (Sponsor mit Adresse**): | Inselspital, Universitätsspital Bern, Freiburgstrasse 8, 3010 Bern |
| **Ort der Durchführung**: | Inselspital, Bern |
| **Verantwortliche Prüfärztin am Studienort:** Name und Vorname in Druckbuchstaben: | Prof. Dr. med. Evangelia Liakoni |
| **Teilnehmende Person:** Name und Vorname in Druckbuchstaben: Geburtsdatum: | weiblich  männlich |

- Ich wurde vom/von der unterzeichnenden Prüfarzt/Prüfärztin mündlich und schriftlich über den Zweck, den Ablauf der Studie, über mögliche Vor- und Nachteile sowie über eventuelle Risiken informiert.
- Ich nehme an dieser Studie freiwillig teil und akzeptiere den Inhalt der abgegebenen schriftlichen Information. Ich hatte genügend Zeit, meine Entscheidung zu treffen.
- Meine Fragen im Zusammenhang mit der Teilnahme an dieser Studie sind mir beantwortet worden. Ich behalte die schriftliche Information und erhalte eine Kopie meiner schriftlichen Einwilligungserklärung.
- Ich bin einverstanden, dass die zuständigen Fachleute des Sponsors und der zuständigen Ethikkommission zu Prüf- und Kontrollzwecken in meine unverschlüsselten Daten Einsicht nehmen dürfen, jedoch unter strikter Einhaltung der Vertraulichkeit.
- Bei Studienergebnissen, die direkt meine Gesundheit betreffen, werde ich informiert.
- Ich kann jederzeit und ohne Angabe von Gründen von der Studienteilnahme zurücktreten. Meine weitere medizinische Behandlung ist unabhängig von der Studienteilnahme immer gewährleistet. Die bis zum Rücktritt erhobenen Daten und Proben werden für die Auswertung zur Studie verwendet.
- Ich bin darüber informiert, dass eine Versicherung Schäden deckt, die auf die Studie zurückzuführen sind.
- Ich bin mir bewusst, dass die in der Studieninformation genannten Pflichten einzuhalten sind. Im Interesse meiner Gesundheit kann mich der/die Prüfarzt/Prüfärztin jederzeit von der Studie ausschliessen.

| Ort, Datum | Unterschrift teilnehmende Person |
| --- | --- |

**Bestätigung der Prüfperson:** Hiermit bestätige ich, dass ich dieser teilnehmenden Person Wesen, Bedeutung und Tragweite der Studie erläutert habe. Ich versichere, alle im Zusammenhang mit dieser Studie stehenden Verpflichtungen gemäss geltendem Recht zu erfüllen. Sollte ich zu irgendeinem Zeitpunkt während der Durchführung der Studie von Aspekten erfahren, welche die Bereitschaft der teilnehmenden Person zur Teilnahme an der Studie beeinflussen könnten, werde ich sie umgehend darüber informieren.

| Ort, Datum | Name und Vorname der Prüfärztin/des Prüfarztes in Druckbuchstaben  Unterschrift der Prüfärztin/des Prüfarztes |
| --- | --- |
