# Supplementary material for: Efficacy of paracetamol added to WHO step III opioids in chronic pain control: study protocol for a randomised, double-blind, placebo-controlled, non-inferiority, multicentre study in Switzerland
Source: BMJ Open. 2025 Dec 31;15(12):e107360. doi: 10.1136/bmjopen-2025-107360 (PMC12766828; doi:10.1136/bmjopen-2025-107360)
Supplement: Supplementary file 2 [file bmjopen-2025-107360.reviewer_comments.pdf]

PEER REVIEW HISTORY

BMJ Open publishes all reviews undertaken for accepted manuscripts. Reviewers are asked to complete a checklist review form and are provided with free text boxes to elaborate on their assessment. These free text comments are reproduced below.

ARTICLE DETAILS

Title (Provisional)

Efficacy of paracetamol added to WHO Step III opioids in chronic pain control: study protocol for a randomized, double-blind, placebo-controlled, non-inferiority, multicenter study in Switzerland

Authors

Kotoula, Christina; Wertli, Maria M.; Streitberger, Konrad; Rothschild, Sacha I; Limacher, Andreas; Hammann, Felix; Krahenbuhl, Stephan; Haschke, Manuel; Liakoni, Evangelia

---

VERSION 1 - REVIEW

**Reviewer**	**1**
**Name**	**Hawley, Philippa**
**Affiliation**	**Univ British Columbia**
**Date**	**22-Aug-2025**
**COI**	**None**

Thanks for the opportunity to review this protocol for a proposed study of withdrawal of acetaminophen in people living with cancer pain. I think it is doable and will be useful. There are some minor suggestions I would like to see considered in the wording however. The reference to the WHO pain ladder should be for CANCER pain, or "chronic cancer pain" at least, not "chronic pain". The approach to non-cancer pain is quite different, opioids being reserved for those that have not responded to non-opioid medications or procedures. This omission is repeated a number of times in the manuscript, including in the patient selection criteria. It needs to be clear that participants will have cancer, and pain will be due to the cancer itself, not to treatment. It will be important to clearly identify the presence of neuropathic pain as well as nociceptive and mixed, with subtype of pain(s) clearly identified. A little more attention to diagnostic processes would be helpful as I suspect they will need to refer back to diagnosis more than the demographic variables at the end. I would also be interested to see if they could ask patients if they have found medical cannabis or cannabis extracts to be helpful at baseline as this may also be a predictor of benefit from

acetaminophen and this would be a good opportunity to explore the potential relationship. If it is available in Switzerland then stable use or non-use should be documented.

The reference to "smart medicine" efforts (line 60) should be explained, as the initiative seems uniquely Swiss, and that term will not always be understood in other countries.

A negative attribute of adjuvant acetaminophen is the pill burden, which is not mentioned in the manuscript.

**Reviewer**	**2**
**Name**	**Rakusa, Martin**
**Affiliation**	**University Medical Centre Maribor**
**Date**	**05-Sep-2025**
**COI**	**None**

The authors propose a well-designed, methodologically strong protocol with high clinical importance. If completed successfully, the trial could directly influence WHO pain management recommendations and prescribing practices worldwide.

There are several major concerns that should be addressed before this manuscript can be suitable for publication.

1. The Authors will include all patients with cancer, regardless of the type or stage. This is problematic because:

a. Heterogeneity in pain mechanisms; pain differs widely between tumour types (e.g., bone metastases vs visceral pain vs. neuropathic infiltration).

b. Disease stage matters; patients with metastases usually require more analgesics.

c. External validity; it isn’t clear to which type of cancer results apply.

2. The Authors also don’t define the type of opioids. Therefore:

a. Opioids substantially differ in potency, pharmacodynamics, and pharmacokinetics.

b. Pooling opioids could obscure paracetamol interactions with specific opioids.

c. The study does not standardise or stratify by opioid dose

3. Taken together, this study will be impossible to replicate.

In addition, the follow-up time of 7 days is very short, and the 8 mm scale is not the most common. I expect at least 28days of follow-up and use of a 10 mm or 12 mm scale.

VERSION 1 - AUTHOR RESPONSE

Reviewer 1:

Thanks for the opportunity to review this protocol for a proposed study of withdrawal of acetaminophen in people living with cancer pain. I think it is doable and will be useful. There are some minor suggestions I would like to see considered in the wording however.

Response
Thank you very much for considering the importance of the project and providing us recommendations to optimize our manuscript.

1) The reference to the WHO pain ladder should be for CANCER pain, or "chronic cancer pain" at least, not "chronic pain". The approach to non-cancer pain is quite different, opioids being reserved for those that have not responded to non-opioid medications or procedures. This omission is repeated a number of times in the manuscript, including in the patient selection criteria. It needs to be clear that participants will have cancer, and pain will be due to the cancer itself, not to treatment.

Response
We thank the reviewer for this comment. The study was originally focused on cancer pain, hence the initial title. Following recruitment challenges and considering that the guidelines apply broadly to pain management, we amended the protocol to also include non-cancer patients with chronic pain. The title and "Study Procedures" section have been revised accordingly for clarity. Hence, we would prefer to keep the wording "chronic pain" throughout the manuscript.

"Efficacy of paracetamol in addition to WHO Step III opioids in chronic pain control: a study protocol for a randomized, double-blind, placebo-controlled, non-inferiority, multicentric study"

"Initially, the study was focused on cancer patients. However, due to recruitment challenges and the current use of WHO guidelines in general pain management in real life clinical setting, we decided to also include non-cancer patients with chronic pain."

2) It will be important to clearly identify the presence of neuropathic pain as well as nociceptive and mixed, with subtype of pain(s) clearly identified.

Response
We would like to thank the reviewer for this comment. Although we previously considered using the DN4 questionnaire together with a physical examination, due to time constraints and after extensive discussions with the study members, we decided to exclude it from the procedures to allow feasibility for the study and facilitate recruitment. At present, we collect information on the main pain diagnosis and localization, comorbidities (which might include e.g. neuropathic pain), medication (including paracetamol dose, name, dose and route of strong opioid, co-analgesics/rescue medications) as well as the use of non-pharmacological measures (e.g. heat/cold application, physiotherapy). We thus hope that some differentiation regarding these aspects will still be possible but we will take your suggestion into consideration and evaluate the possibility of implementing such an assessment in a future amendment.

3) A little more attention to diagnostic processes would be helpful as I suspect they will need to refer back to diagnosis more than the demographic variables at the end.

Response

Currently, we are collecting information on main pain diagnosis, pain localization, comorbidities, current medication list, possible risk factors for hepatotoxicity, vital signs, several questions regarding pain (including localization and nonmedical measures to relieve pain) and for cancer patients we additionally document the type of cancer, month and year of its first diagnosis, metastases and their localization and additional cancer treatment (e.g. chemotherapy, radiation, chemotherapy). A more detailed documentation regarding the diagnosis would most probably not be feasible due to reporting limitations and would also lead to visits taking much more time thus leading to recruitment problems if patients are not willing to spend so much time at the study center. For clarity, we revised the sentence describing the diagnostic processes.

"The study team will collect the demographic data, medical and pain history (including - if applicable - cancer type and time of first diagnosis of cancer, metastases, length of chronic cancer pain treatment prior to study, other cancer treatments (e.g. chemotherapy, radiotherapy)), main pain diagnosis, pain localization, pain duration/percentage time with pain pro 24 hours), comorbidities, medication (including paracetamol dose, name, dose and route of strong opioid used in the 7 days prior to study begin and any potential co-analgesics/rescue medication), smoking status…"

4) I would also be interested to see if they could ask patients if they have found medical cannabis or cannabis extracts to be helpful at baseline as this may also be a predictor of benefit from acetaminophen and this would be a good opportunity to explore the potential relationship. If it is available in Switzerland then stable use or non-use should be documented.

Response
We thank the reviewer for this valuable comment. Although we do not specifically assess participants' perceptions or preferences regarding medical cannabis or cannabis extracts (not the focus of the study), all concomitant medications (fixed and rescue) are documented at baseline, and participants are asked at days 7 and 14 about any changes since their previous visit (see Table 2). Thus, any medically taken cannabis products should be included in the documentation and concomitant medications and their indications will be considered in the data analysis. Moreover, we ask for any drug use at the screening visit and the pain diary includes a section for non-medical measures, thus also non-medical use of cannabis should be adequately recorded and could be investigated as a predictor in the data analysis.

5) The reference to "smart medicine" efforts (line 60) should be explained, as the initiative seems uniquely Swiss, and that term will not always be understood in other countries.

Response
Following an editor's comment, this section has been revised and the reference to "smart medicine" was deleted:

- A randomized, double-blind, placebo-controlled, multicentre study to assess the efficacy of paracetamol when combined with strong opioids in a larger population with chronic pain.
- Inclusion of cancer and non-cancer patients, reflecting real-world practice.
- Inclusion of genotyping of relevant opioid receptors and CYP enzymes, along with

measurement of paracetamol and opioid blood concentrations.

- Recruitment challenges such as shifts in prescription patterns or patients avoiding additional visits beyond routine care

6) A negative attribute of adjuvant acetaminophen is the pill burden, which is not mentioned in the manuscript.

Response
As recommended, we have revised and included "pill burden" in the section "Clinical implications".

"Thus, removing unnecessary paracetamol prescriptions and reducing the pill burden could meaningfully improve patient safety and outcomes."

Reviewer 2:

The authors propose a well-designed, methodologically strong protocol with high clinical importance. If completed successfully, the trial could directly influence WHO pain management recommendations and prescribing practices worldwide. There are several major concerns that should be addressed before this manuscript can be suitable for publication.

Response
Thank you very much for recognizing the importance of our study and for taking the time to provide feedback on our manuscript.

1) The Authors will include all patients with cancer, regardless of the type or stage. This is problematic because:
a. Heterogeneity in pain mechanisms; pain differs widely between tumour types (e.g., bone metastases vs visceral pain vs. neuropathic infiltration).
b. Disease stage matters; patients with metastases usually require more analgesics.
c. External validity; it isn't clear to which type of cancer results apply.

Response
We acknowledge that pain may vary across tumor types, cancer stages and other comorbidities. However, the World Health Organization (WHO) guidelines do not differentiate recommendations by cancer type or stage and are currently applied broadly in both cancer and non-cancer pain management. Further, patients that will be included are under a constant pain medication use and thus, in a stable condition. Consequently, we anticipate heterogeneity not only between cancer subgroups but also between cancer and non-cancer patients. To address this, we will collect detailed information on pain characteristics (diagnosis, location, duration, and (if applicable) cancer type, stage and metastasis) and perform subgroup analyses. With a larger sample size than previous studies, this approach will allow us to generate insights into the role of paracetamol in the combination with strong opioids across diverse patient groups, reflecting real-world clinical practice.

2) The Authors also don't define the type of opioids. Therefore:
a. Opioids substantially differ in potency, pharmacodynamics, and pharmacokinetics.

b. Pooling opioids could obscure paracetamol interactions with specific opioids.
c. The study does not standardize or stratify by opioid dose

Response
In this study, we plan to include only co-medication with strong opioids, those are defined in the Design and study procedures section ("…patients receiving a strong opioid (i.e. morphine, oxycodone, methadone, fentanyl, hydromorphone, or buprenorphine)…"). At screening we document the type, dose and frequency of opioids and rescue medications. Standardizing opioid doses would have required dose adjustments (e.g. reduction) which could have destabilized analgesia and confounded interpretation of paracetamol withdrawal and rescue medication use. Instead of stratifying by opioid dose, we stratify by baseline pain level, as pain is the primary outcome and a direct determinant of treatment response, while opioid dose itself could be influenced by pain severity. As described in the statistical analysis section, we will additionally account for the specific opioid used, its dose and concentrations and perform subgroup analyses where appropriate. Moreover, genotyping for opioid receptors and metabolizing enzymes will allow us to further explore interindividual variability in opioid dosing, concentrations and response. Hence, while multiple strong opioids are included, their pharmacological differences are systematically addressed through detailed documentation, genetic analyses and planned subgroup analyses.

3) Taken together, this study will be impossible to replicate.

Response
We hope that our responses have addressed your concerns. While some heterogeneity is expected, this is consistent with other pragmatic clinical studies. To ensure replicability, we carefully document all relevant data and plan subgroup analyses to account for potential variability, thereby providing results that reflect real-world clinical practice.

4) In addition, the follow-up time of 7 days is very short, and the 8 mm scale is not the most common. I expect at least 28days of follow-up and use of a 10 mm or 12 mm scale.

Response
We thank the reviewer for this comment. The scale used for the primary outcome is a 100 mm visual analogue scale (VAS), not 8 mm. A difference of > 8 mm on the 100 mm scale is considered clinically relevant. Further details on the sample size estimation are provided in the section "Estimated sample size and power." If we were to define a clinically relevant difference as 10 mm or 12 mm, the required sample size would be substantially reduced. This means that, theoretically, we will have sufficient data to also analyze the results under the assumption that a larger difference represents greater clinical relevance in practice.

Regarding study duration, we acknowledge that the initial design included 20 days (10 days/ phase). Due to recruitment challenges and patients feedback that 10 days without paracetamol would be too long, particularly for cancer patients many of whom were in advanced stages where every day is significant, we reduced the duration to 14 days (7 days/ phase) to enhance feasibility.

VERSION 2 - REVIEW

Reviewer 2

Name Rakusa, Martin

Affiliation University Medical Centre Maribor

Date 07-Nov-2025

COI

The main concern regarding the proposed study design is sample heterogeneity. The authors indicate that they intend to address this by "collecting detailed information on pain characteristics (diagnosis, location, duration, and cancer type, stage, and metastasis) and performing subgroup analyses." However, to ensure that such subgroup analyses are statistically meaningful, the authors should provide a clear sample size calculation that accounts for these planned subgroups.

A second issue concerns the rationale and consistency regarding pain medication use. The authors state that "patients included are under constant pain medication use and thus in a stable condition," yet they also mention that "patients reported that 10 days without paracetamol would be too long." These two statements appear contradictory and require clarification.

To make the study design acceptable for publication, the authors should:

Provide a transparent sample size calculation, explicitly considering subgroup analyses.

Clarify whether patients are in a stable condition with respect to pain medication use and management.

VERSION 2 - AUTHOR RESPONSE

Reviewer 2:

1) The main concern regarding the proposed study design is sample heterogeneity. The authors indicate that they intend to address this by "collecting detailed information on pain characteristics (diagnosis, location, duration, and cancer type, stage, and metastasis) and performing subgroup analyses." However, to ensure that such subgroup analyses are statistically meaningful, the authors should provide a clear sample size calculation that accounts for these planned subgroups.
Provide a transparent sample size calculation, explicitly considering subgroup analyses.

Response:
The sample size was calculated for the primary outcome measure, i.e. average pain intensity on day 7 measured using a VAS, which represents the main objective of the trial. All the additional analyses (e.g. secondary outcomes and subgroup analyses) will be exploratory to identify potential patterns. The study is not powered to detect statistically significant effects within each subgroup or secondary outcome. However, all the statistical analyses for primary and secondary outcomes are pre-specified in the section "Statistical analysis". Furthermore, the randomization is expected to provide a balancing effect in the heterogeneous population.

We have now clarified the sample size calculation and also comment on the heterogeneity in the revised manuscript:
"For the randomization, which is expected to provide a balancing effect in the heterogeneous population by balancing known and unknown factors between the groups…"

"Secondary outcomes and subgroup analyses will be investigated in an exploratory manner and no sample size or power calculations have been performed for these analyses."

2) A second issue concerns the rationale and consistency regarding pain medication use. The authors state that "patients included are under constant pain medication use and thus in a stable condition," yet they also mention that "patients reported that 10 days without paracetamol would be too long." These two statements appear contradictory and require clarification. Clarify whether patients are in a stable condition with respect to pain medication use and management.

Response:
We thank the reviewer for his comment. For clarification: The "stable condition" refers to the analgesic treatment (not to the clinical condition) *prior* to the study, which has to be stable for at least seven days in order for the patient to be eligible (Table 1: Eligibility criteria). The other statement refers to feedback received from some patients regarding the time *post* start of study treatment, during which some might have a blinded withdrawal of paracetamol (phase 1 of the study) followed by a non-blinded withdrawal (phase 2 of the study). This is not part of the inclusion and exclusion criteria but rather represents the belief of some patients that paracetamol shows efficacy in this combination, which is the study question we want to investigate (especially the second part of the study should provide valuable information regarding the placebo effect).
However, due to recruitment challenges and this feedback from potential participants indicating this as the reason for not wanting to participate in the study, we shortened the total duration to 7 days per study phase. We hope this clarifies that participants must have stable analgesic treatment prior to randomization and that the only change in their treatment is the discontinuation of paracetamol after randomization. For clarification, we adapted the manuscript as follows:

"The participants will start with the study medication (paracetamol or placebo) at the same paracetamol dose previously used directly after their screening/baseline visit and will stop the study medication on day 7. During the whole study duration, the patients are allowed to continue with any other analgesics stable prescribed prior to study inclusion. **This ensures that their overall analgesic treatment remains unchanged to pre-randomization, except substitution of paracetamol with placebo in one of the two groups after randomization and stop of paracetamol/placebo during the second study phase."**

VERSION 3 - REVIEW

**Reviewer**	**2**
**Name**	**Rakusa, Martin**
**Affiliation**	**University Medical Centre Maribor**

Date 15-Dec-2025

COI

Authors responded to comments. The manuscript may be accepted.
